# Dynamic neutrophil-to-lymphocyte ratio predicts prognosis in patients with soft tissue sarcoma: a retrospective study of 231 cases

**DOI:** 10.3389/fonc.2026.1792397

**Published:** 2026-04-21

**Authors:** Yong Jiang, Yongli Ding, Weibing Peng, Mingming Zhao, Longqing Li, Ge Li, Yongzhou Luo, Xinchang Lu

**Affiliations:** 1 Orthopaedic Department, The First Affiliated Hospital of Henan University of Chinese Medicine, Zhengzhou, Henan, China; 2 Department of Orthopaedic Surgery, The First Affiliated Hospital of Zhengzhou University, Zhengzhou, Henan, China

**Keywords:** delta neutrophil-to-lymphocyte ratio (delta-NLR), hematological biomarker, prediction, prognosis, soft tissue sarcoma

## Abstract

**Background:**

Soft tissue sarcomas (STS) exhibit significant heterogeneity and are classified as rare tumors with a high risk of metastasis. The neutrophil-to-lymphocyte ratio (NLR), a hematological marker indicative of systemic inflammation, has gained broad recognition for its prognostic utility in oncology. This ratio can be used to evaluate the dynamic changes in inflammatory markers during the diagnosis and treatment of tumors. The value of NLR fluctuations in STS has yet to be fully investigated.

**Methods:**

This investigation involved a retrospective cohort of 231 patients with STS, all definitively diagnosed and managed at the Musculoskeletal Tumor Center of The First Affiliated Hospital of Zhengzhou University, aiming to evaluate their clinical profiles. The research focused on analyzing the impact of both baseline NLR and its dynamic changes throughout therapy on the prognostic outcomes in STS, with the aim of constructing a nomogram based on delta-NLR.

**Results:**

The study cohort comprised 231 individuals diagnosed with STS. Based on delta-NLR trends, participants were categorized into two cohorts: an NLR increase group (n=94) and an NLR decrease group (n=137). Analysis using time-dependent receiver operating characteristic (ROC) curves revealed that delta-NLR possessed greater predictive accuracy for prognosis relative to other hematologic parameters and clinical characteristics. Both univariate and multivariate analyses determined that Fédération Nationale des Centres de Lutte Contre le Cancer (FNCLCC) grade, patient age, and delta-NLR served as independent predictors of prognosis. A prognostic nomogram was subsequently constructed integrating these significant factors. The nomogram achieved a C-index of 0.702, and calibration curves verified its accuracy in predicting three- and five-year overall survival (OS) for STS patients. Results from decision curve analysis (DCA) and clinical impact curve assessment additionally validated that utilizing this delta-NLR-based nomogram may offer substantial clinical utility in the management of STS.

**Conclusion:**

NLR is valuable for continuous monitoring, and ongoing assessment of NLR provides better survival predictions for patients with STS than using baseline NLR alone.

## 1 Introduction

Soft tissue sarcomas (STS) refer to a complex group of malignant cancers of mesenchymal origin, which occupy about 1% of all malignancies and exhibit high risk of metastasis (1). Owing to the high rate of recurrence, the wide spectrum of pathological subtypes, pronounced biological specificity, and marked tissue heterogeneity, the diagnosis and management of soft tissue sarcomas remain highly challenging (2). With the standard treatment strategy combining surgical resection, radiotherapy, and chemotherapy, the prognosis of STS has improved. However, over half of patients with STS develop local recurrence or distant metastasis following extensive surgical resection (3), and the 5-year survival rate remains below 30% (4). Currently, aside from chemotherapy, anti-angiogenesis agents, and a few specific targeted therapies, effective treatment options for advanced, metastatic, and recurrent patients with STS are still limited (5). Therefore, timely recognition of high-risk factors for recurrence or distant metastasis in patients with STS is essential for optimizing treatment strategies and disease management (4). At present, disease progression prediction is primarily based on clinical and pathological factors, including diagnostic age, tumor size, histological grading, tissue subtype, tumor depth, tumor site, and margin status (6). However, the diagnostic process for such lesions heavily relies on the subjective experience of physicians, with a relatively high risk of false-positive misjudgments. As such, there is an urgent need in clinical practice to establish an efficient and simple prognostic assessment system to stratify recurrence risks for patients with STS and provide individualized adjunctive treatments and follow-up schedules.

Recent advances in tumor biomarkers have centered on proteins, genetic and epigenetic alterations, and liquid biopsy technologies (7–10). The high cost and operational complexity of STS treatments have remained major obstacles to widespread clinical implementation. In contrast, inflammation—an established hallmark of cancer—plays a pivotal role in tumor initiation and progression (11), and inflammatory markers are increasingly being incorporated into patient stratification and clinical outcome prediction (12). As a representative hematological indicator of systemic inflammation, the neutrophil-to-lymphocyte ratio (NLR) has gained recognition for its prognostic value in oncology (13–15). NLR can reflect the crosstalk between chronic inflammation and the immune response (16). An elevated NLR has been associated with poorer disease-free survival (DFS) and overall survival (OS), as well as a higher risk of locoregional recurrence (LRR) (17, 18). Notably, most current studies continue to rely exclusively on baseline NLR assessments, which are inadequate for capturing dynamic changes in tumor progression throughout treatment (19, 20). Chemotherapy-associated factors, including intratumoral heterogeneity and drug resistance, can alter a patient’s inflammatory profile, thereby highlighting the potential of dynamic hematological indicators for capturing the real-time biological behavior of tumors (21, 22). Therefore, evaluating the dynamic changes in inflammatory markers throughout the entire course of tumor diagnosis and treatment holds significant clinical value. In hepatocellular carcinoma, colorectal cancer, and osteosarcoma, the prognostic value of dynamic NLR changes has been validated (23–25). For example, dynamic NLR monitoring provides superior predictive performance for survival compared with baseline NLR in osteosarcoma patients (25). Nevertheless, although emerging evidence suggests that NLR fluctuations during treatment may offer more accurate prognostic insights in cancer patients (26, 27), their value in soft tissue sarcoma has yet to be fully validated.

Thus, we proposed using delta-NLR as a prognostic indicator for STS to reflect tumor changes during treatment, aiming to improve tumor detection and patient prognosis. This study retrospectively analyzed the dynamic changes in NLR levels preoperatively and postoperatively in patients, aiming to preliminarily validate the prognostic significance of NLR dynamics in STS.

## 2 Patients and methods

### 2.1 Patients

**Figure 1:**
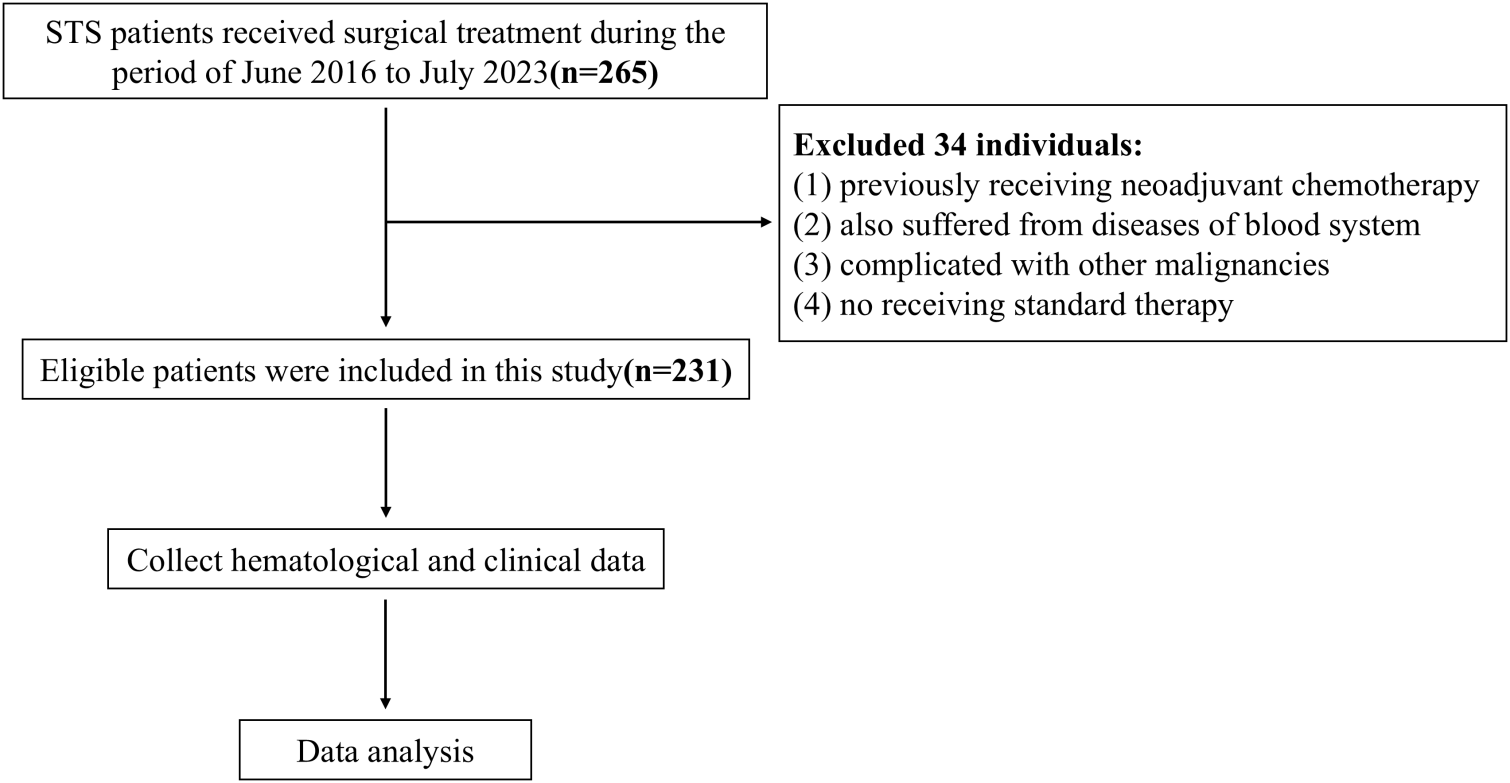
Flowchart of patient selection and the study design.

The retrospective study included STS patients admitted to the Musculoskeletal Tumor Center, the First Affiliated Hospital of Zhengzhou University, between June 2016 and July 2023. The inclusion criteria were as followed: First, patients with a pathological confirmation of STS; Second, patients who had complete hematological test results from our hospital; Third, patients who received standard treatment at our center. The exclusion criteria were as followed: First, patients who had been subjected to neoadjuvant chemotherapy prior to initial consultation at our center; Second, Patients with hematological disorders; Third, Patients with concomitant malignant tumors; Fourth, Patients who failed to receive standard treatment. A total of 231 patients were enrolled, with regular follow-up conducted for each patient until death or July 2023. The follow-up rules adhered to that of previous studies (25). The flowchart of the study design is shown in **Figure 1**. The study obtained the approval of the Ethics Committee of the First Affiliated Hospital of Zhengzhou University, and acquired participants’ written informed consent. The ethics committee approval number is 2025-KY-1986.

### 2.2 Data collection and analysis

Hematological biomarkers were collected from the 231 STS patients’ primary blood routine before operation. NLR was computed using neutrophil count and lymphocyte count at the following time points, which were designated as baseline NLR (NLR1) and postoperative NLR (NLR2), calculated by considering the results of the first complete blood count (CBC) prior to any treatment, and the last CBC within 90 days after surgery, respectively (28). Subtracting baseline NLR from postoperative NLR yielded the Delta−NLR values, which were stratified into two groups: higher or lower. Notably, data from patients who had received leukocyte growth factors (e.g., recombinant human granulocyte colony-stimulating factor) were excluded via the electronic medical record system. Additionally, we collected as well as analyzed relevant clinical data of age, gender, body mass index (BMI), Fédération Nationale des Centres de Lutte Contre le Cancer (FNCLCC) grade, tumor size, and tumor location, and calculated the OS from the date when patients were diagnosed to the date when they died or the last follow-up date.

### 2.3 Primary outcomes

The study held the primary objective of exploring the relationship among NLR, delta-NLR, and OS in STS patients undergoing standard treatment. We defined OS as the time from the initiation of treatment to death, and patients who remained alive at the final follow-up from the final analysis. Besides, the association and interplay between these indexes were also elucidated.

### 2.4 Statistical analysis

The Kolmogorov-Smirnov test was employed to verify the normality of continuous variables. According to the normality results, the study applied either the t-test or the Mann-Whitney U test for the difference comparison in continuous variables, and the chi-squared test or Fisher’s exact test for categorical variables, taking into account the sample size of each group. All statistical analyses relied on R software (version 4.4.0; Vienna Institute of Statistics and Mathematics, Austria). P < 0.05 denoted statistical significance.

## 3 Results

### 3.1 Patients demographics

The cohort comprised 231 STS patients with 110 males and 121 females. The mean age were 45.0 ± 18.3 years (range 12–89). FNCLCC grade categorized 166 patients as stage 3 and 65 as stage 2. Tumor distribution was as follows: upper limbs (n=39), lower limbs (n=152), and trunk (n=40). Tumor size stratification ascertained 33 patients with lesions <5 cm, 113 patients with 5–10 cm tumors, and 85 patients with tumors >10 cm. At the last follow-up, 51 patients were recorded as deaths and the mean OS was 52.3 ± 28.3 months (**Table 1**).

**Table 1:** Patients demographics.

	Patients	delta-NLR		P-value
		Increase	Decrease	
Total patients	231	94	137	
Overall survival				
Mean (SD)		1245(776)	1824(821)	< 0.001
Age(years)				
Mean (SD)	45.0(18.3)	49.2(18.3)	42.3(17.6)	0.005
Gender				
Male	110	46	64	
Female	121	50	71	
FNCLCC				0.009
Stage 2	65	20	45	
Stage 3	166	76	90	
Location				0.341
Upper extremity	39	13	26	
Lower extremity	152	64	88	
Trunk	40	19	21	
Tumor Size(cm)				0.200
T<5 cm	33	10	23	
5 cm<T<10 cm	113	46	67	
T>10cm	85	40	45	
BMI (kg/m^2^)				0.879
Abnormal	74	31	43	
Normal	157	65	92	

### 3.2 Establishment and validation of the delta-NLR in STS

**Figure 2:**
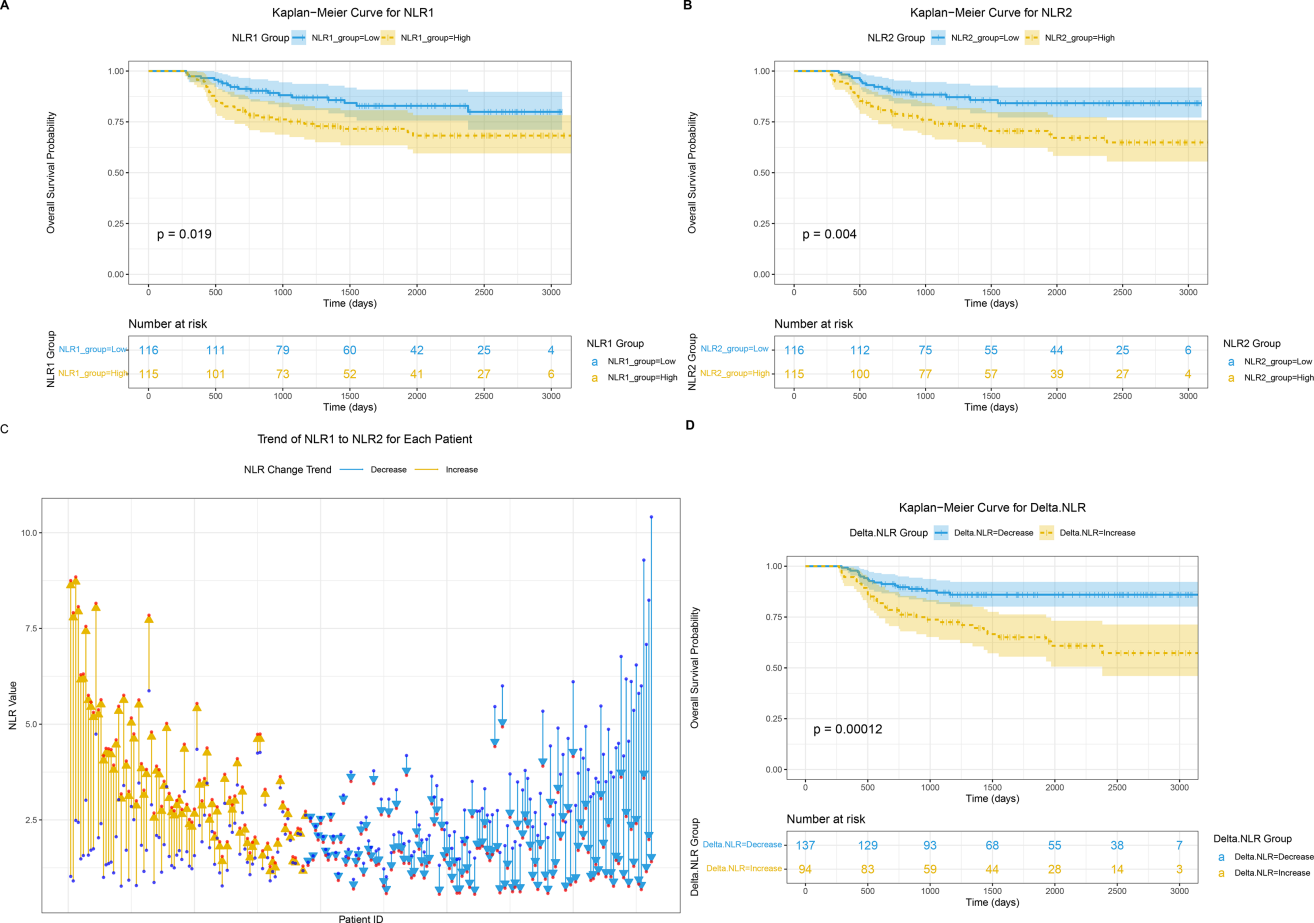
Kaplan-Meier (KM) survival curves illustrate OS of patients stratified by **(A)** NLR1, **(B)** NLR2, and **(D)** Delta NLR. **(C)** The Trend of NLR1 to NLR2 for Each Patient. (The neutrophil-to-lymphocyte ratio (NLR) was computed using neutrophil count and lymphocyte count. Baseline NLR (NLR1) and postoperative NLR (NLR2), calculated by considering the results of the first complete blood count (CBC) prior to any treatment, and the last CBC within 90 days after surgery, respectively. Subtracting baseline NLR from postoperative NLR yielded the Delta−NLR values).

231 patients with STS were stratified by hematological biomarker grades. Compared with the high NLR1 group, patients in the low NLR1 group had better survival (P = 0.019; **Figure 2A**). Similarly, the low NLR2 group showed superior survival than the high NLR2 group (P = 0.004; **Figure 2B**), and the low delta-NLR group had significantly better survival than the high delta-NLR group (P = 0.00012; **Figure 2D**). **Figure 2C** depicts the dynamic changes of NLR in each patient during treatment: most patients had fluctuating NLR, with more showing a decreasing trend than an increasing trend, consistent with the finding that delta-NLR-Decrease was more common than delta-NLR-Increase.

### 3.3 Prognostic predictive ability of hematological markers

**Figure 3:**
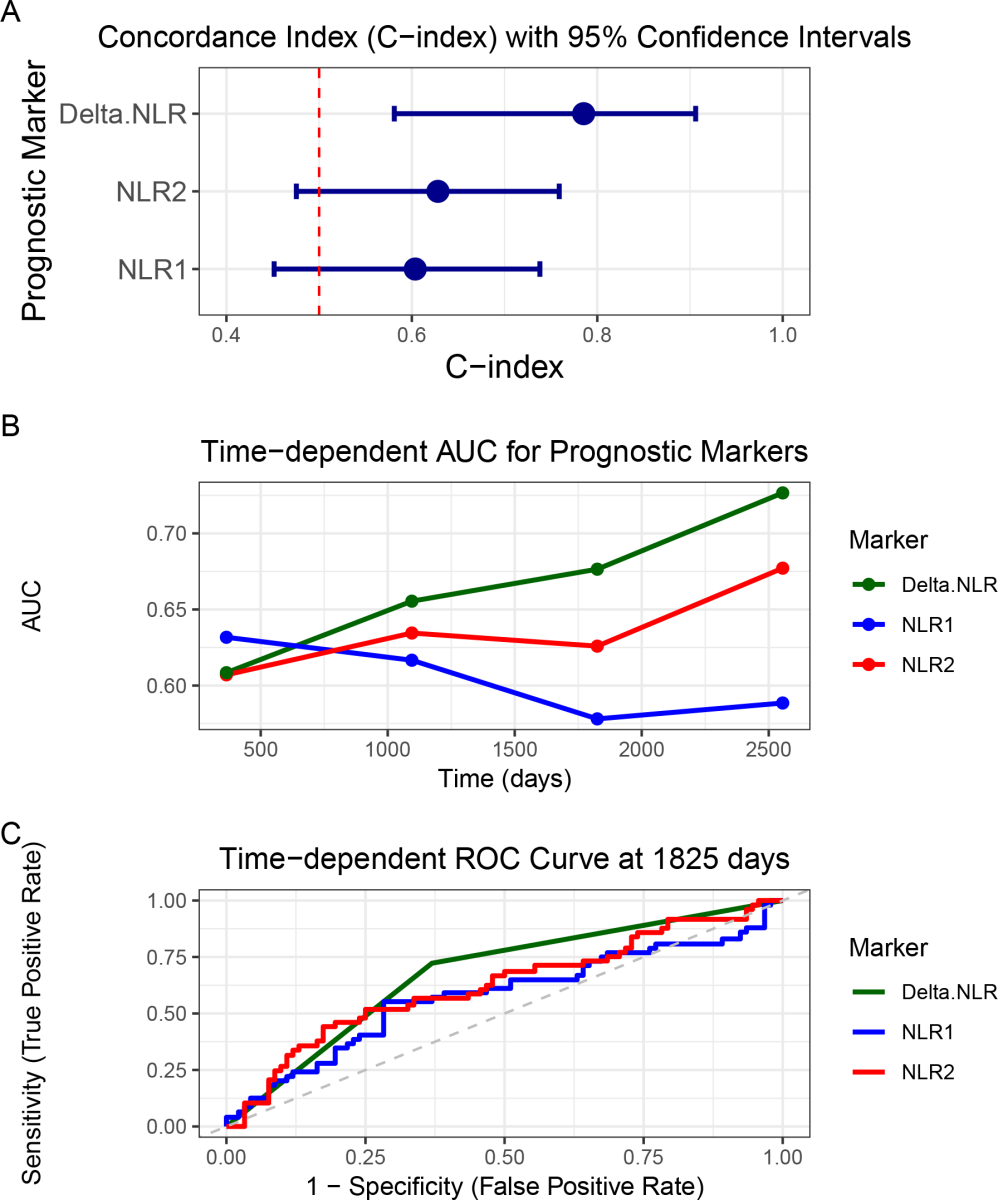
**(A)** The forest plot demonstrates the prognostic predictive performance of various prognostic markers. **(B)** Time-dependent Area Under the Curve (AUC) results demonstrate differences in prognostic predictive performance among various prognostic markers. **(C)** Time-dependent Receiver Operating Characteristic (ROC) curves depict the predictive sensitivity of STS prognostic factors and reflect changes in their predictive performance, with a higher AUC value indicating superior prognostic predictive ability for STS.

**Figure 4:**
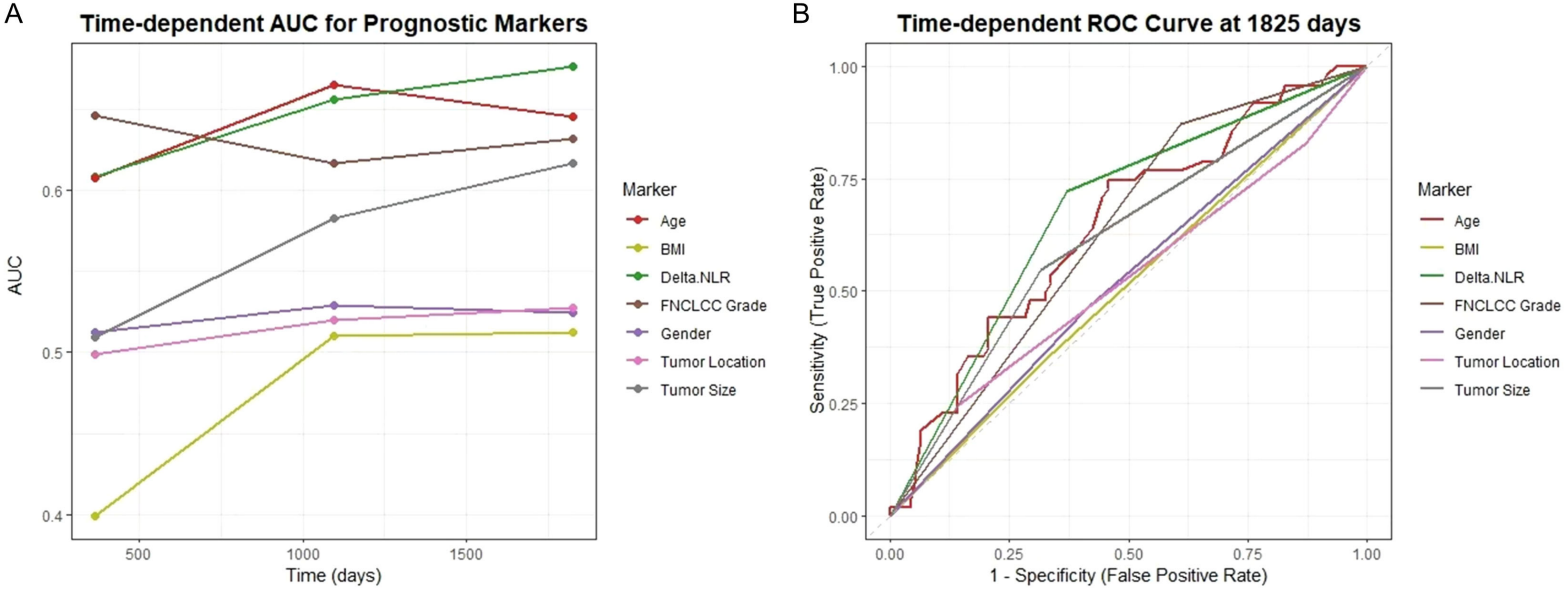
**(A)** Time-dependent AUC result illustrates the variances in predictive capabilities of different prognostic markers. **(B)** The predictive sensitivity of STS prognostic factors is depicted by time-dependent ROC curves.

Cox regression models were performed to investigate the relationship between hematological markers and OS. As shown in **Figure 3A**, all markers exhibited C-indices above 0.5, indicating their prognostic value. Notably, delta-NLR achieved a mean C-index of 0.8, substantially higher than that of NLR2 and NLR1 (both 0.6). Additionally, delta-NLR showed a narrower 95% CI, demonstrating superior prognostic accuracy and result reliability compared to NLR2 and NLR1 (**Figure 3A**). As shown, a larger AUC in the delta-NLR than that of other markers including NLR1 and NLR2 was observed in the time−dependent receiver operating characteristic (ROC) curve (**Figure 3B**). The delta-NLR curve consistently remains at the highest level. Overall, the sensitivity and specificity of delta-NLR are optimal at 1825 days, which indicates that its predictive efficacy is also the best (**Figure 3C**). The AUC of delta-NLR significantly increases with extended follow-up time, suggesting that its predictive efficacy for long-term prognosis gradually strengthens (**Figure 4A**). In conjunction with **Figure 3C**, the sensitivity and specificity of delta-NLR for predicting survival rates are optimal at the 5-year mark, while Age and FNCLCC grade demonstrate comparable predictive efficacy in terms of sensitivity and specificity (**Figure 4B**).

### 3.4 Univariate analysis and multivariate analysis

**Figure 5:**
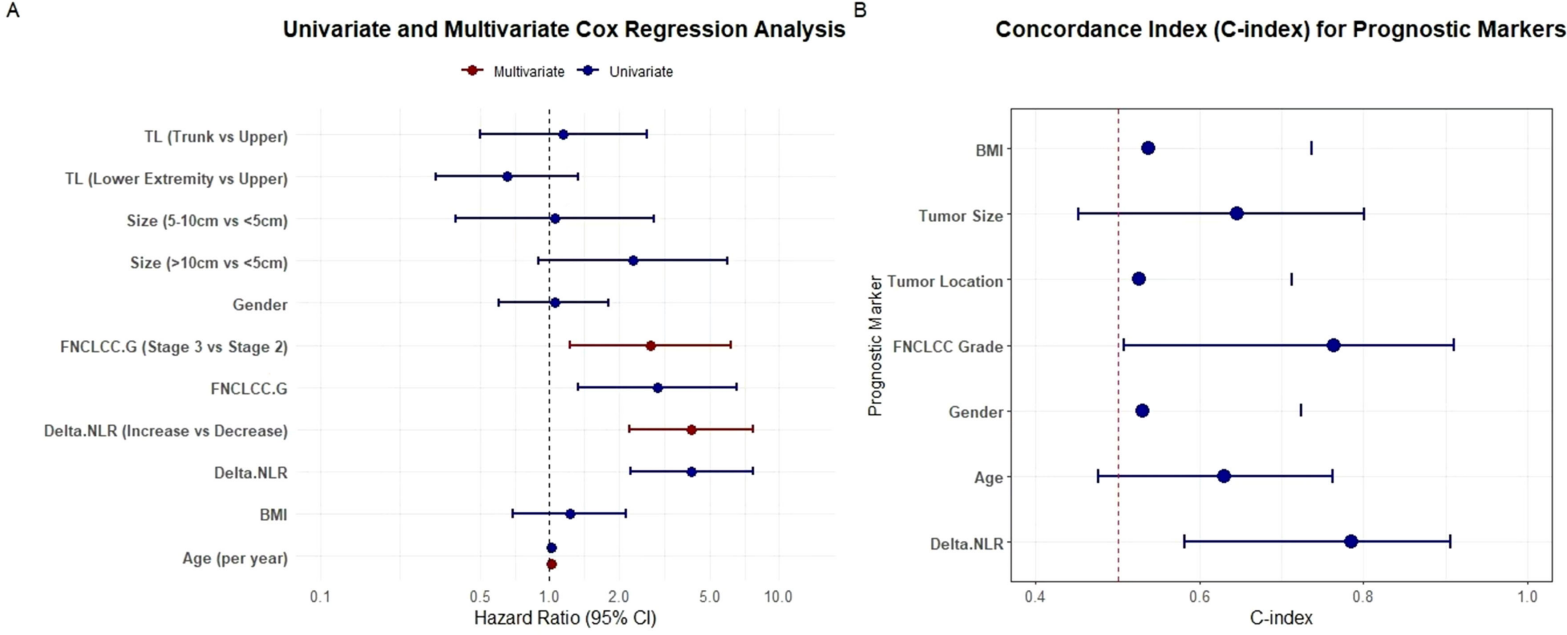
**(A)** Univariate and multivariate analyses of clinical characteristics and hematological biomarkers. **(B)** Forest plot showing the prognostic predictive performance of various prognostic markers.

Univariate and multivariate analyses were conducted among 231 patients to further explore the prognostic value of variables in STS. Univariate analysis revealed that FNCLCC grade and delta-NLR were correlated with OS. Multivariate analysis was then performed to identify independent prognostic risk factors for OS, which indicated that FNCLCC grade (Stage 3 vs. Stage 2) and delta-NLR (Increase vs. Decrease) were independent risk factors for STS (**Figure 5A**). C-index results indicated that FNCLCC grade and delta-NLR were significantly correlated with OS in patients with STS. FNCLCC grade and delta-NLR were both effective in predicting OS; however, delta-NLR exhibited a higher C-index, indicating superior predictive performance (**Figure 5B**).

### 3.5 Construction and validation of a delta-NLR-based nomogram

**Figure 6:**
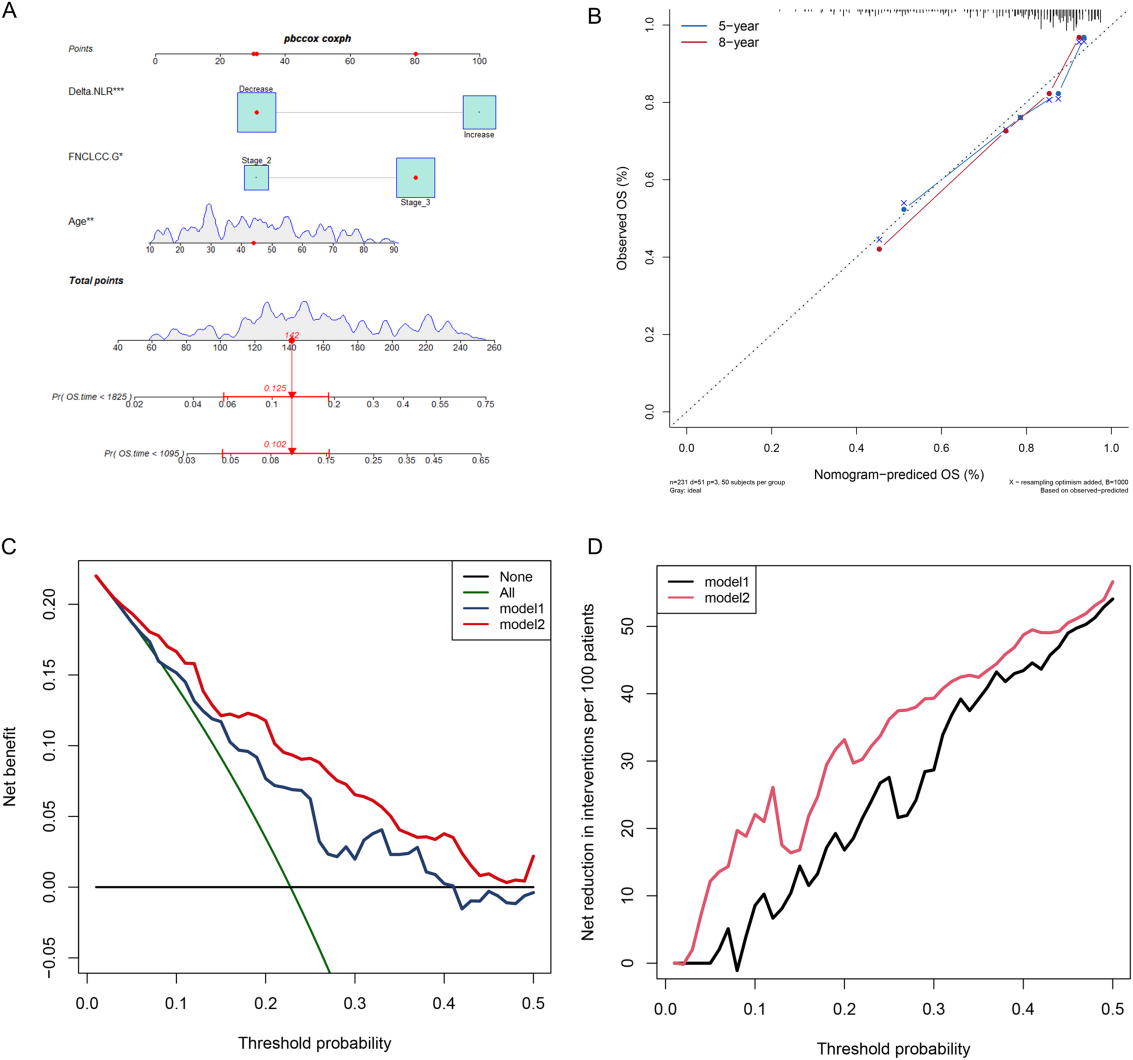
Construction and validation of the STS overall survival nomogram. **(A)** Nomogram constructed by integrating delta-NLR, Fédération Nationale des Centres de Lutte Contre le Cancer (FNCLCC) grade, and age; the total nomogram score was the sum of individual covariate scores. **(B–D)** Validation of the nomogram via calibration curve, decision curve analysis, and clinical impact curve. (Subtracting baseline NLR from postoperative NLR yielded the Delta−NLR values).

We constructed a nomogram integrating delta-NLR with clinical characteristics that exhibited improved clinical applicability. The Cox proportional hazards regression model was used to assign a score to each covariate according to its hazard ratio, and the total nomogram score was calculated as the sum of these individual scores (**Figure 6A**). The C-index of this STS-specific nomogram was 0.702, and the calibration curve confirmed its effectiveness in predicting three- and five-year overall survival rates in patients with STS (**Figure 6B**). Furthermore, a decision curve analysis (DCA) was performed to evaluate the clinical utility of this nomogram (**Figures 6C, D**). The results showed that the nomogram incorporating delta-NLR offered significant net benefits compared to the model containing only clinical characteristics.

## 4 Discussion

We retrospectively enrolled 231 STS patients to systematically identify independent factors influencing long-term survival and preliminarily validate the predictive value of delta-NLR. Multivariate analysis indicated that delta-NLR is an independent adverse prognostic factor for STS, with superior discriminative ability compared to conventional clinical or laboratory parameters. Moreover, by integrating delta-NLR with key clinical variables, we constructed a nomogram that effectively predicted three-year and five-year survival outcomes in patients with STS. Thus, we conclude that delta-NLR is a reliable tool for individualized prognostic evaluation in patients with STS.

STS represent a rare, highly heterogeneous group of malignancies, encompassing over 80 distinct histological subtypes and accounting for roughly 1% of all human cancers (29). More than half of patients with STS develop local recurrence or distant metastasis following surgical resection (30), and the median survival time for those with distant metastases is 1 to 1.5 years (31). In clinical practice, prognostic assessment primarily relies on key indicators, including tumor location, size, metastatic status, histological subtype, pathological grade, and the Enneking staging system (32). Our preliminary findings demonstrated that the Lung Immune Prognostic Index (LIPI) offered superior predictive performance for the prognosis of patients with STS compared to conventional clinical indicators (15).

Tumor-associated inflammation is a key factor in remodeling the tumor microenvironment (TME), and its correlation with STS has been thoroughly investigated (33, 34). Inflammation-related indices, including NLR, the platelet-to-lymphocyte ratio (PLR), serum lactate dehydrogenase (LDH), and LIPI, have predictive significance for OS in patients with gastric cancer, osteosarcoma, and STS (15, 25, 35, 36). Previous researches have shown that an elevated NLR indicates active systemic inflammation. This microenvironment is closely associated with poor prognosis in cancer patients through mechanisms such as the production of pro-angiogenic factors, inhibition of pro-apoptotic signaling pathways, induction of DNA damage, and acceleration of circulating tumor cell proliferation (37, 38). Our preliminary study confirmed that NLR holds value in continuous monitoring. Compared to baseline NLR, persistently monitored NLR can better predict survival outcomes in patients with osteosarcoma (25).

While previous studies have predominantly focused on baseline NLR, emerging evidence indicates that dynamic changes in NLR during treatment provide greater reference value for prognostic assessment than other prognostic predictors (39–43). NLR is used to compare the extent of inflammatory and immune responses, reflecting the relative intensity of the host’s inflammatory and immune reactions (44). delta-NLR, or the change in NLR, was defined as the variation between the baseline NLR and the NLR measured after treatment (45). Traditionally, NLR has been assessed at a single time point before treatment, and few studies have investigated post-treatment NLR changes as a prognostic marker (46). The ability of dynamic changes in NLR to enhance patient management has been established in hepatocellular carcinoma and osteosarcoma (25, 45). Nonetheless, the potential of the delta-NLR to predict the prognoses of patients with STS remains indeterminate. Hence, this study was the first to explore the correlation between delta-NLR and STS, and constructing a delta-NLR-based prognostic model targeting STS. The LIPI outperformed clinical markers (age and FNCLCC grade) in the prediction of STS patients’ long-term survival. Delta-NLR could further categorize patients into two distinct risk levels, enhancing their prognostic risk stratification and assisting in treatment selection. Moreover, the time−dependent ROC curves demonstrated that the delta-NLR had a better prognostic ability than NLR1 and NLR2, suggesting that this comprehensive index offers greater benefits compared to using a single hematological inflammation index. The delta-NLR-based nomogram may facilitate OS prediction in patients with STS and thereby enable more effective treatment and follow-up strategies (**Figure 6**). For example, the points for Δ−NLR, age, and stage III were 30, 32, and 80, respectively, with a total score of 142. A total score of 142 corresponds to a 5−year survival rate of approximately 87.5% and a 3−year survival rate of approximately 89.8%. Therefore, more frequent follow-ups and active intervention are recommended for such patients to improve long-term survival. Based on the delta-NLR scoring system for STS patients, targeted management measures coupled with follow-up protocols can be formulated to implement targeted management.

This study acknowledges limitations that suggest promising directions for future inquiry. First, the single-center study may have introduced bias. Nevertheless, the cohort comprised 231 STS patients, and was one of the largest studies as we know. Therefore, the findings may reasonably reflect the efficacy of delta-NLR in the prediction of STS patients’ prognosis, necessitating the adoption of a multicenter approach to more deeply assess the predictive model’s efficacy in STS cases in future research. In our future studies, we will enroll a larger sample size and perform external validation to further investigate the prognostic predictive ability of Δ−NLR in patients with STS. Second, the retrospective nature of this study may have induced recall bias. Because of the rarity and heterogeneity of STS, it is difficult to conduct prospective studies on the prognostic prediction in patients with STS. In future studies, we plan to expand the sample size via multicenter collaboration by enrolling more STS patients, so as to conduct subtype stratification analyses with greater statistical power and further validate and clarify the prognostic predictive ability of Δ−NLR in different subtypes. In future research, we plan to employ time-dependent Cox models or landmark analysis in a larger cohort to further optimize the statistical strategy and enhance the robustness of our conclusions. In summary, this finding requires further validation by multicenter, large−sample, and prospective studies.

## 5 Conclusion

This study evaluated the utility of delta-NLR in predicting the prognosis of 231 patients with STS. Delta-NLR was identified as an independent risk factor for patients with STS. The nomogram constructed based on delta-NLR may help clinicians predict the OS of patients with STS, thereby facilitating timely intervention and personalized clinical management.

## Data Availability

The raw data supporting the conclusions of this article will be made available by the authors, without undue reservation.
